# Palliative interatrial septum stenting with a vascular stent in a chronic thromboembolic pulmonary hypertension patient: Is it beneficial?

**DOI:** 10.1186/s43044-023-00397-8

**Published:** 2023-08-12

**Authors:** Swastya Dwi Putra, Radityo Prakoso, Aditya Agita Sembiring, Damba Dwisepto Aulia Sakti, Suko Adiarto, Arwin Saleh Mangkuanom, Yovi Kurniawati

**Affiliations:** 1 Department of Cardiology and Vascular Medicine, Faculty of Medicine, Universitas Indonesia, DKI Jakarta, 11420 Indonesia; 2 National Cardiovascular Center, Harapan Kita, Letjen S. Parman Kav. 87, West Jakarta, DKI Jakarta, 11420 Indonesia

**Keywords:** Interatrial septum stenting, CTEPH, Vascular stent, Atrial septostomy, Case report

## Abstract

**Background:**

Chronic thromboembolic pulmonary hypertension (CTEPH) is a serious disease that can progress and lead to a deadly outcome. Despite optimal drug therapy, pulmonary hypertension (PH) remains fatal. Untreatable right heart failure (RHF) from CTEPH is eventually a significant cause of death. However, unloading the right heart and increasing systemic output are the treatment goals in these patients.

**Case presentation:**

A 42-year-old female presented to the emergency department with worsening dyspnea experienced for three days before admission. There were also complaints of leg edema, ascites, orthopnea, and palpitation. Physical examination revealed an attenuated second heart sound, abdominal ascites, and bilateral leg edema. She had a history of frequent readmissions due to RHF despite optimal medical therapy and was diagnosed with CTEPH 5 months ago. It was decided that the patient would undergo interatrial septal (IAS) stenting with a vascular stent of 8 mm × 39 mm × 135 cm. The results were good; her symptoms and signs of RHF improved, and she was eventually discharged from the hospital. Four months after the procedure, the patient was able to engage in physical activities without any limitations.

**Conclusions:**

A palliative IAS stent is one of the choices for intractable RHF management in patients with CTEPH. The vascular stent can be used as an alternative in order to make the interatrial connection more stable and last longer.

## Background

CTEPH is a serious disease that can be fatal for a patient. Even optimal drug therapy has failed to change that. Untreatable RHF from CTEPH is a significant cause of death [1]. However, unloading the right heart and increasing systemic output are the treatment goals in these patients. Atrial septostomy (AS) is an interventional procedure serving this purpose but with the limitation of spontaneous closure at later follow-ups. Nowadays, an atrial flow regulator (AFR) device is used for unloading the right ventricle in advance of PH and is useful to prevent spontaneous closure. Unfortunately, the availability of AFR is limited in several countries. Thus, it is important to seek an alternative approach, such as using a vascular stent [2], since it makes a better and more stable interatrial connection. This review aims to describe a case of palliative IAS stenting using a vascular stent in a CTEPH patient.

## Case presentation

**Fig. 1:**
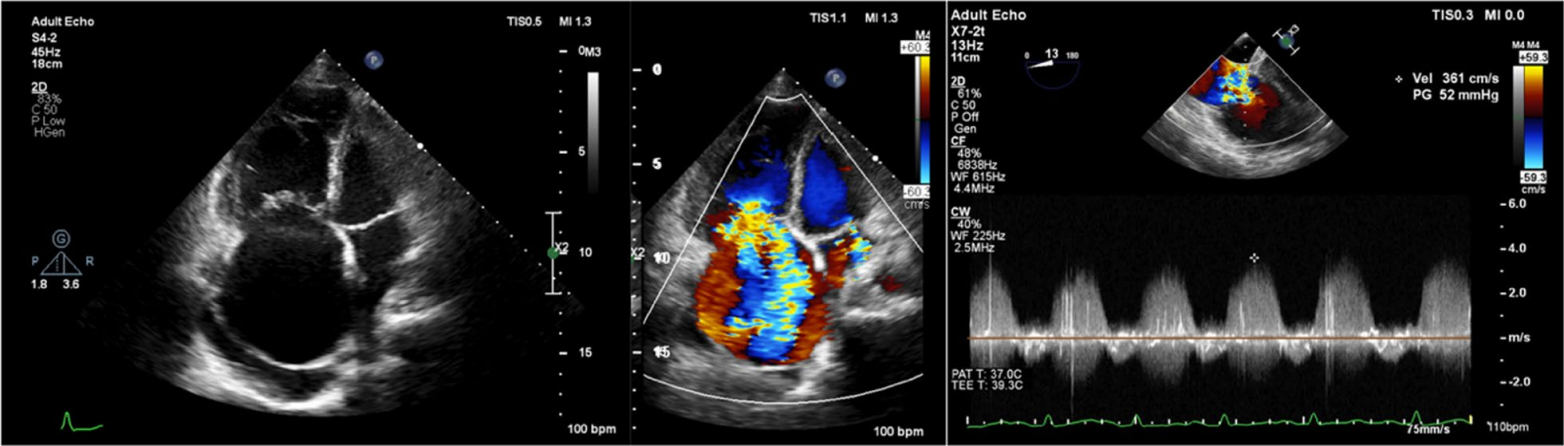
Echocardiography showed dilation of the right atrium and ventricle, bulging IAS to the left atrium, and severe tricuspid valve regurgitation

A 42-year-old female presented to the hospital with dyspnea, leg edema, ascites, orthopnea, and palpitations that she had been experiencing for three days. She had a history of frequent readmissions due to RHF, despite optimal medical therapy. She was diagnosed with CTEPH 5 months ago by computed tomography pulmonary artery (CTPA). Her CTPA revealed the presence of peripheral subsegmental thrombus in parts of the apical anterior upper lobes bilaterally and the right anterior-posterobasal lower lobes. Physical examination showed oxygen saturation of 90%, blood pressure of 96/69 mmHg, an attenuated second heart sound, a pansystolic murmur of grade 3/6 on the lower left sternal border, abdominal ascites, and bilateral leg edema. Electrocardiography showed sinus rhythm, right atrial enlargement, and right bundle branch block. Laboratory results were within normal limits. The chest X-ray showed cardiomegaly, right atrial enlargement, and right ventricle hypertrophy. Echocardiography showed severe tricuspid regurgitation (TR) with a high probability of PH, paradoxical septal wall motion, the interatrial septum bulging to the left side, decreased right ventricle contractility with tricuspid annular plane systolic excursion (TAPSE) of 16 mm, and no patent foramen ovale (PFO) (Fig. 1). Thus, the patient was diagnosed with RHF due to CTEPH. She received sildenafil 3 × 40 mg, warfarin 1 × 3 mg, candesartan 1 × 4 mg, and spironolactone 1 × 25 mg.

**Fig. 2:**
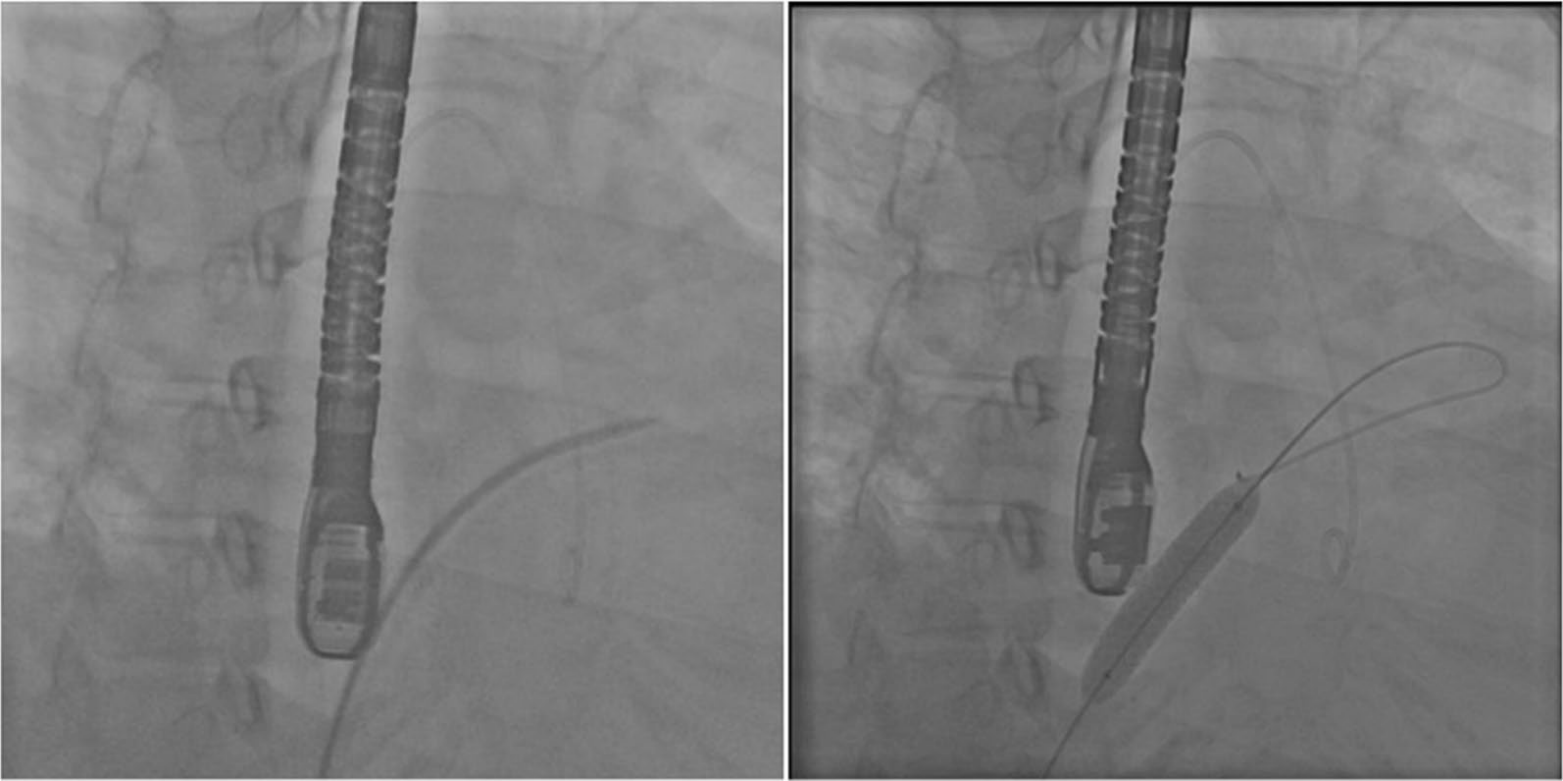
Atrial septostomy with Brockenbrough needle and IAS stent with vascular stent

**Fig. 3:**
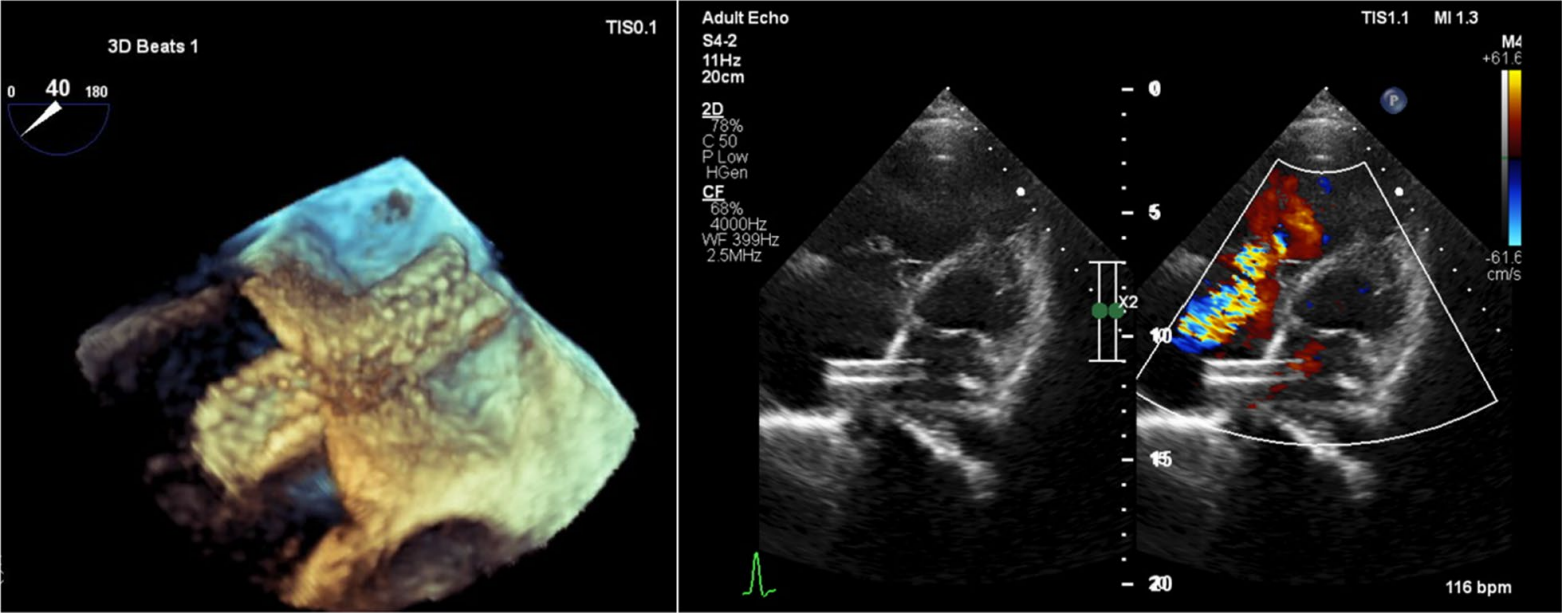
The position of the IAS stent as seen by echocardiography

The surgical procedure could not be done because the patient refused to undergo the procedure, and pulmonary thromboendarterectomy is rarely performed in our country. Thus, it was decided that the patient would undergo an atrial septostomy with an IAS stent. Prior to the procedure, the patient underwent right heart catheterization revealed right atrial (RA) pressure of 20 mmHg and left atrial (LA) pressure of 9 mmHg, obtained through left ventricular end-diastolic pressure (LVEDP) measurements as a surrogate of LA pressure. Cardiac output before the procedure was obtained using the direct method of Fick’s formula and resulted in 2.7 L/min. A Mullin catheter was inserted through the right femoral vein, and an atrial septostomy was done using a Brockenbrough needle. Before placing the stent, graded dilatation was carried out using a 4 × 40 mm thysak balloon. After this procedure, the oxygen saturation dropped to 85%. A vascular stent, the Omnilink Elite, measuring 8 mm by 39 mm by 135 cm, was placed at the IAS (Fig. 2). After the procedure, right heart catheterization was repeated, which revealed RA pressure of 28 mmHg and LA pressure of 10 mmHg, obtained from measurements taken through an atrial septostomy. LVEDP after the procedure was 12 mmHg. Cardiac output after the procedure was obtained using the direct method of Fick’s formula and was 3.5 L/min. Transthoracic and transesophageal echocardiography evaluation revealed a good stent position that IAS was in the middle third of the stent to avoid stent dislodgement, with a right-to-left shunt across the stent (Fig. 3). Peripheral saturation after the procedure was 79%.

In the cardiovascular care unit, the patient received optimal heart failure therapy with an inotropic, diuretic, and vasodilator. The patient was eventually discharged with improved RHF signs and symptoms. Before being discharged, she underwent a 6-min walking test, and walked 120 m, compared to 50 m before the procedure, when she could not walk more than 50 m and spent more time in bed. Four months after the procedure, the patient was in good condition, with a peripheral saturation of 82%, and could perform physical activities without any limitations with a Minnesota Living with Heart Failure Questionnaire (MLHFQ) score of 22.

## Discussion

CTEPH is a major cause of chronic PH, which leads to RHF and death. This condition can lead to insufficient blood flow to the left cardiac chambers and decreased cardiac output. Pulmonary hypertension drugs may improve the prognosis of the patient, such as sildenafil, bosentan, riociguat, and macitentan; however, not all patients can have access to these kinds of treatments due to their limited availability in developing countries, and riociguat is not available in our country. Moreover, not all patients respond well to these therapies. Atrial septostomy, Potts shunt, pulmonary thromboendarterectomy, and lung transplantation are various choices for treating drug-refractory PH [1, 2].

Patients in the WHO functional classes III-IV with right heart failure unresponsive to adequate pharmacological therapy or with significant syncopal symptoms are considered appropriate for AS treatment according to ESC/ERS recommendations for diagnosing and treating pulmonary hypertension [3]. The rationale of AS in CTEPH is to create and maintain atrial septal communication with the right-to-left shunt so it decompresses the right cardiac chambers and increases the preload of the left chambers without developing severe hypoxemia [4].

A study by Gorachevsky SV, et al. examined PH patients with intermediate and high risk who underwent IAS stents. They found that PH patients with high risk have a higher mortality rate than those with moderate risk. Thus, they suggest that those undergoing IAS stents are patients with intermediate risk [5]. However, in the series, Sandoval J et al. described about 62% of patients not getting PAH-specific treatment before AS. The authors do not recommend delaying the intervention until medical treatment is no longer effective. They postulate that an intervention that is carried out at a relatively early stage of the disease would have promising outcomes [4].

In this case, our patient was included as a high-risk patient because of severe right ventricular failure that was refractory to optimal medical therapy. The patient received sildenafil for the treatment of PH because that was the only oral pulmonary artery dilator available in Indonesia. However, we remained committed to performing an atrial septostomy and IAS placement to improve her quality of life.

However, the AS technique has some drawbacks, one of which is spontaneous closure at later follow-up that necessitates additional procedures in these critically ill patients [5, 6]. Placing a device, like an AFR, in this patient is useful to prevent this spontaneous closure and create interatrial communication. Unfortunately, the availability of AFR is limited in several countries, such as Indonesia. Thus, it is important to seek another device for an alternative approach; we need a large-size stent to make a good interatrial mix, for instance, using a vascular stent. The key to properly placing an IAS stent is that the IAS must be positioned in the middle third of the stent to avoid stent dislodgement. Unsheathing the distal half of the stent within the left atrium and partially expanding only the distal left atrial end help to obtain a stable dog-bone configuration. Then, this sheath-stent-balloon assembly is withdrawn until the expanded left atrial end is caught and resisted by the atrial septum. This confirms the location of the middle of the stent against the atrial septum. Once this point is reached, the stent-balloon assembly is fully unsheathed. A small flush of contrast from the sidearm of the transseptal sheath will confirm the location of the stent in relation to the atrial septum [6, 7].

In our case, we used a vascular stent because we needed a larger diameter than a coronary stent and used a sheathless technique for delivering the stent to its location in the atrial septum. The stent was inflated when the IAS was in the middle third of the stent, with fluoroscopy and echocardiography guidance. The procedure was done with good results, and she was eventually discharged with improved RHF signs and symptoms.

## Conclusions

Palliative IAS stenting is one of the alternative approaches for decompressing the RV in intractable RHF. This treatment provides stable interatrial communication that can last longer than the results of conventional treatments.

## Data Availability

Not applicable.

## Abbreviations

AFR: Atrial flow regulator
AS: Atrial septostomy
CTEPH: Chronic thromboembolic pulmonary hypertension
CTPA: Computed tomography pulmonary artery
IAS: Interatrial septum
MLHFQ: Minnesota Living with Heart Failure Questionnaire
PFO: Patent foramen ovale
PH: Pulmonary hypertension
RHF: Right heart failure
TAPSE: Tricuspid annular plane systolic excursion
